# Somatostatin Receptor PET/CT Imaging for the Detection and Staging of Pancreatic NET: A Systematic Review and Meta-Analysis

**DOI:** 10.3390/diagnostics10080598

**Published:** 2020-08-16

**Authors:** Matteo Bauckneht, Domenico Albano, Salvatore Annunziata, Giulia Santo, Priscilla Guglielmo, Viviana Frantellizzi, Alessia Branca, Cristina Ferrari, Antonio Vento, Alessia Mirabile, Anna Giulia Nappi, Laura Evangelista, Pierpaolo Alongi, Riccardo Laudicella

**Affiliations:** 1 Nuclear Medicine Unit, IRCCS Ospedale Policlinico San Martino, 16132 Genoa, Italy; matteo.bauckneht@gmail.com; 2 Department of Nuclear Medicine, University of Brescia and Spedali Civili Brescia, 25123 Brescia, Italy; doalba87@libero.it; 3 Nuclear Medicine Unit, IRCSS Regina Elena National Cancer Institute, 00168 Rome, Italy; salvatoreannunziata@live.it; 4 Nuclear Medicine Unit, Department of Interdisciplinary Medicine, University of Bari Aldo Moro, 70121 Bari, Italy; giuliasanto92@gmail.com (G.S.); alessia9130@gmail.com (A.B.); ferrari_cristina@inwind.it (C.F.); anna.giulia.nappi@gmail.com (A.G.N.); 5 Nuclear Medicine Unit, AO Brotzu, 09134 Cagliari, Italy; priscilla.guglielmo@yahoo.it; 6 Department of Molecular Medicine, Sapienza University of Rome, 00185 Rome, Italy; viviana.frantellizzi@uniroma1.it; 7 Department of Biomedical and Dental Sciences and of Morpho-Functional Imaging, Nuclear Medicine Unit, University of Messina, 98125 Messina, Italy; antvento@alice.it (A.V.); alessia.mirabile86@gmail.com (A.M.); riclaudi@hotmail.it (R.L.); 8 Nuclear Medicine Unit, Department of Medicine-DIMED, University of Padova, 35128 Padova, Italy; 9 Unit of Nuclear Medicine, Fondazione Istituto G.Giglio, 90015 Cefalù, Italy; alongi.pierpaolo@gmail.com

**Keywords:** pancreas, neuroendocrine tumors, positron emission tomography, somatostatin receptor analogs

## Abstract

We investigated the diagnostic performance of Somatostatin Receptor Positron Emission Tomography/Computed Tomography (SSR-PET/CT) for the detection of primary lesion and initial staging of pancreatic neuroendocrine tumors (pNETs). A comprehensive literature search up to January 2020 was performed selecting studies in presence of: sample size ≥10 patients; index test (i.e., 68Ga-DOTATOC or 68Ga-DOTANOC or 68Ga-DOTATATE PET/CT); and outcomes (i.e., detection rate (DR), true positive, true negative, false positive, and false-negative). The methodological quality was evaluated with QUADAS-2. Pooled DR and pooled sensitivity and specificity for the identification of the primary tumor were assessed by a patient-based and a lesion-based analysis. Thirty-eight studies were selected for the qualitative analysis, while 18 papers were included in the meta-analysis. The number of pNET patients ranged from 10 to 142, for a total of 1143 subjects. At patient-based analysis, the pooled sensitivity and specificity for the assessment of primary pNET were 79.6% (95% confidence interval (95%CI): 71–87%) and 95% (95%CI: 75–100%) with a heterogeneity of 59.6% and 51.5%, respectively. Pooled DR for the primary lesion was 81% (95%CI: 65–90%) and 92% (95%CI: 80–97%), respectively, at patient-based and lesion-based analysis. In conclusion, SSR-PET/CT has high DR and diagnostic performances for primary lesion and initial staging of pNETs.

## 1. Introduction

The incidence of pancreatic neuroendocrine tumors (pNETs) is less or equal to one case per one hundred thousand people per-year, and they account for roughly 5% of all pancreatic cancers. However, in the last few decades, their incidence has risen [1,2].

Many biological features make these tumors clinically heterogeneous, including mutational status [3,4], hormone production, and histopathological grade. Among them, NETs grading, which is mainly related to Ki-67 expression and mitotic index, has several diagnostic, therapeutic, and prognostic implications. In well-differentiated pNETs (G1, G2, and well-differentiated G3) the slow cancer growth is related to good long-term survival even in the presence of liver metastases [5], while poorly differentiated G3 neuroendocrine carcinomas (NECs) show higher proliferation rates and lower overall survival [6]. Moreover, tumor grade is strictly related to the expression of somatostatin receptors (SSR1-5) on the neoplastic cellular surface [6,7]. In low-grade pNETs, the high SSR expression allows the therapeutic use of somatostatin analogs and makes these neoplasms ideal for targeted radionuclide imaging [8]. In contrast, the down-regulation of SSR makes high-grade NEC less suitable for these approaches.

Currently, three radio-labelled somatostatin analogs are used in the clinical practice for targeted SSR radionuclide imaging of pNETs: 68Ga-DOTATATE (DOTA, Tyr(3)-octreotate), 68Ga-DOTANOC (DOTA,1-Nal(3)-octreotide), and 68Ga-DOTATOC (DOTA, D-Phe1, Tyr (3)-octreotide). Although these positron-emitting radiotracers have a different affinity to the various types of SSR [9], they showed a similar diagnostic accuracy [10,11].

Targeted SSR molecular imaging with positron emission tomography and computed tomography (PET/CT) plays a significant role in pNETs clinical management, particularly in the staging phase. Indeed, surgery is the only curative treatment approach, and an accurate assessment of both tumor detection and disease widespread is of utmost importance to avoid unsuccessful procedures. In the present systematic review and meta-analysis, we investigate the diagnostic performance of SSR-PET/CT for the detection of the primary lesion and initial staging of pNETs. 

## 2. Materials and Methods

### 2.1. Literature Search

Four researchers (G.S., A.B., A.M., and A.V.) performed a bibliographic analysis until 1 January 2020, by including PubMed, Scopus, Embase, and Google Scholar databases. The terms “pancreas”, “neuroendocrine”, “NET”, “Positron Emission Tomography”, “Positron Emission Tomography/Computed Tomography”, “68Gallium”, “DOTA”, “somatostatin receptor”, “staging”, “diagnosis”, and “detection” were used for the bibliographic search, in each database. Additional filters, such as English language, original article and/or research article, and study including only humans, were used. Reviews, clinical reports, meeting abstracts, and editor comments were excluded. Four independent reviewers (A.G.N., V.F., P.G., and C.F.) evaluated the full texts of the selected papers. Furthermore, to improve the selection of the papers, the references of the studies included were assessed and included in the research strategy. 

The systematic review was carried out using the standard methods [12], and graphically showed in accordance with the PRISMA guidelines [13]. All studies that fulfilled the inclusion criteria were considered eligible for the systematic review and meta-analysis: (a) a sample size ≥10 patients; (b) 68Ga-DOTATOC, 68Ga-DOTANOC, or 68Ga-DOTATATE PET/CT as index tests; (c) detection rate (DR), true positive (TP), true negative (TN), false positive (FP), and false-negative (FN), which allowed us to construct 2 × 2 contingency tables. In case of studies from the same group of researchers, only the report with the highest number of enrolled patients was considered for the meta-analysis.

### 2.2. Quality Evaluation and Statistical Analysis

The overall quality of the studies was assessed by using the Quality Assessment of Diagnostic Accuracy Studies tool (QUADAS-2) [14]. Meta-Analyst (version Beta 3.13) [15] and Comprehensive meta-analysis software were used to carry out the meta-analysis. Heterogeneity was tested using the χ2 and the I2 tests. The heterogeneity was considered low, moderate, or high in case of a value equal to 25%, 59%, and 75%, respectively [12]. The meta-analysis was carried out with the random-effects model, in accordance with the recommendation of the Cochrane Oral Health Group. The pooled detection rate was calculated for the identification of the primary pNETs. Pooled sensitivity, pooled specificity, positive and negative likelihood ratio (LR+ and LR−), and diagnostic odds ratio (DOR) with 95% confidence intervals (95%CIs) for the evaluation of primary pNET were also computed. Patient-based and lesion-based analyses were carried out. Publication bias was assessed using a funnel plot. 

## 3. Results

### 3.1. Search Results

**Figure 1:**
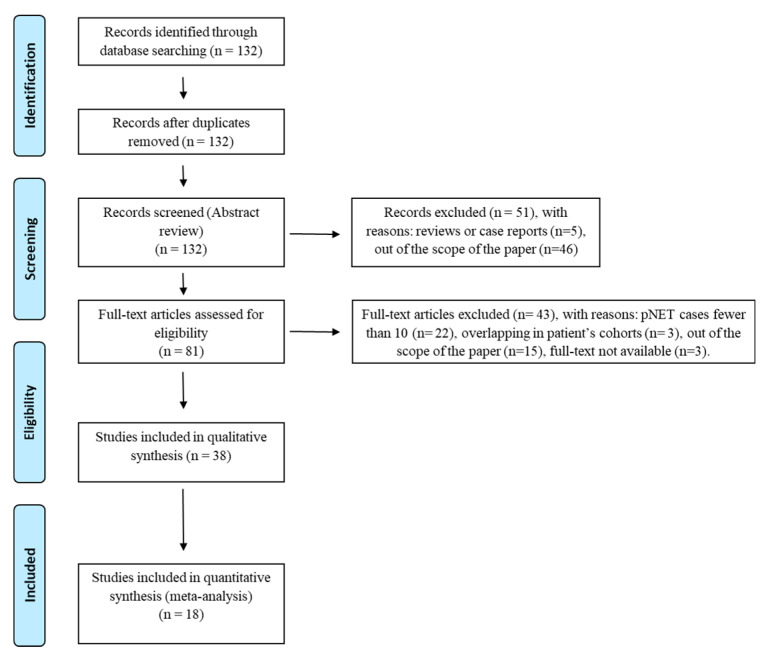
PRISMA flow-chart. Selection process of studies included in the qualitative and quantitative analysis according to the PRISMA flow diagram [13].

The literature search revealed 132 articles. Reviewing titles, abstracts, and full texts, we excluded 94 articles. Therefore, 38 studies were selected and included in the qualitative analysis, while 18 articles were considered for the meta-analysis (Figure 1).

### 3.2. Study Characteristics

**Figure 2:**
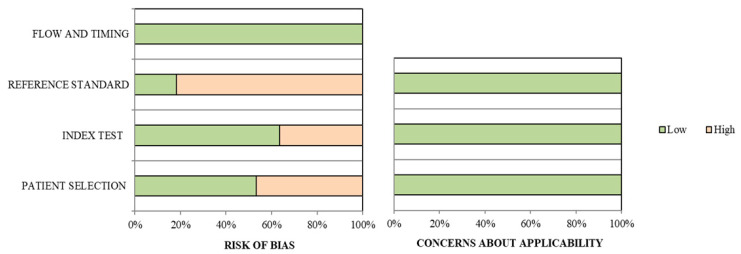
Study quality assessment. Overall quality assessment of the studies included in the systematic review according to QUADAS-2 tool [14].

The clinical and technical characteristics of included studies are detailed in Table 1 and Table 2, respectively, while the study quality assessment is reported in Figure 2. Selected articles were published by researchers from Europe, USA, and Asia. Twenty-two studies were retrospective and 16 studies were prospective. The number of enrolled pNET patients ranged from 10 to 142, and a total of 1143 pNET patients were included. The mean and median age of the patients ranged from 40 to 65 years. Among studies that analyzed tumor grading (*n* = 18), early grading (G1-G2) was more frequent than advanced (G3), despite deriving this data for pNET was not possible. SSR-PET/CT imaging was performed exclusively in the staging or preoperative setting in 18 studies. While most studies were conducted on pNET patients irrespectively to the tumor function, the study by Nockel et al. [16] was focused on patients affected by insulinoma. Similarly, a few studies were focused on hereditary pNETs, including Von Hippel-Lindau (VHL) syndrome [17,18] and Multiple Endocrine Neoplasia Type 1 (MEN-1) [19,20,21].

PET/CT was employed without CT contrast media injection in all research papers except for the studies by Mayerhoefer et al. [32] and by Kazmierczak et al. [45], whereas no studies employed hybrid PET/magnetic resonance imaging (MRI) scanner.

All three SSR-targeted tracers were employed (68Ga-DOTATOC in 13, 68Ga-DOTANOC in seven, and 68Ga-DOTATATE in 14, respectively), while mixed tracers were used in two studies [38,40]. Only in the study by Poeppel et al. [10], two of these radiotracers (68Ga-DOTATOC and 68Ga-DATOTATE) were compared in terms of diagnostic accuracy, showing that maximal uptake of 68Ga-DOTATOC tended to be higher than its 68Ga-DOTATATE counterpart, thus encouraging the application of different SSR ligands in order to personalize imaging and therapy. Similarly, in one study [40], 68Ga-labeled SSR imaging was compared to 64Cu-labeled SSR-PET on a per-lesion and per-patient basis, showing promising results for 64Cu-labeled SSR, both for diagnostic and therapeutic implications.

The injected radiopharmaceutical activity was similar across all studies (Table 2). In contrast, considerable variability in the time interval between radiotracer injection and image acquisition was observed (ranging 20–162 min). However, in the paper by Nakamoto et al. [47], in which two acquisition time points (60 and 90 min post-injection) were tested, no relevant differences in terms of accuracy were reported in either the detection of primary lesion nor in the metastasis identification. Analysis of PET images was exclusively performed using visual analysis in 10 studies [21,23,24,25,28,32,39,42,44,49]. Additional semiquantitative criteria, mainly maximum standardized uptake value (SUVmax), were also used. 

### 3.3. Qualitative Analysis

Most of the selected studies used conventional imaging as the reference standard to assess SSR-PET/CT accuracy in the detection of primary lesions [17,21,24,25,26,27,28,29,30,32,35,41,46,50]. Histopathology was used as a reference only by Cingarlini et al. [48] and Kaemmerer et al. [38]. In the former study, SSR-PET/CT showed high sensitivity (equal to 94.3%) in detecting the primary lesion of G1-2 pNETs [48]. In the latter [38], the authors used the histopathological reference combined with reverse transcriptase-quantitative polymerase chain reaction gene-expression data to evaluate the correlation between Dotapeptides accumulation and SSR2A expression in both primary and metastatic pNET lesions. Reported data showed that SUVmax and SUVmean are reliable ex vivo parameters for in vivo quantification of SSR expression in pNET. 

Considering the studies that evaluated semiquantitative parameters in pNETs [17,19,20,29,30,33,34,35,36,38,41,43,48,51], average lesion SUVmax was 25.4 (range 2–191). Of note, high heterogeneity in SUVmax between the primary lesion and distant metastases was observed. While some studies reported higher SUVmax values for the primary lesion [29,30], in some others [41] the opposite finding was observed. Moreover, a variable site-specific diagnostic accuracy was observed for metastatic lesions when SSR-PET/CT was compared with conventional imaging as the reference standard [22,23,27,28,29,30,32,41,44,46,50,51]. In most studies, SSR-PET/CT was superior to CT for the detection of lymph node, bone, liver, and other organ metastases, while CT overcame SSR-targeted imaging for the assessment of pulmonary metastases.

In a few studies, the diagnostic potential of SSR-PET/CT imaging was also measured in terms of impact on the therapeutic decisions [26,27,28,39,42,50]. Considering all patient cohorts of these studies (*n* = 1513), SSR-PET/CT influenced the therapeutic plan in 597 cases, resulting in a management change in approximately 39% of patients. However, only the study by Ilhan et al. [39] specifically addressed this point in a study cohort of pNET patients, in which imaging results altered the surgical management in 6/18 cases (33%), while the remaining studies contained mixed NET patients′ cohorts. In contrast, fewer studies explored the prognostic relevance of SSR-targeted imaging at staging in pNETs [20,52]. Majala et al. [52] showed that 68Ga-DOTANOC integrated with 18F-Fluorodeoxyglucose (FDG) PET/CT can predict tumor grade and clinical outcome in non-functioning pNETs. The authors showed that not only FDG but also 68Ga-SSR imaging has prognostic value in the preoperative setting, being able to predict the histopathological grade. 

### 3.4. Quantitative Results

**Figure 3:**
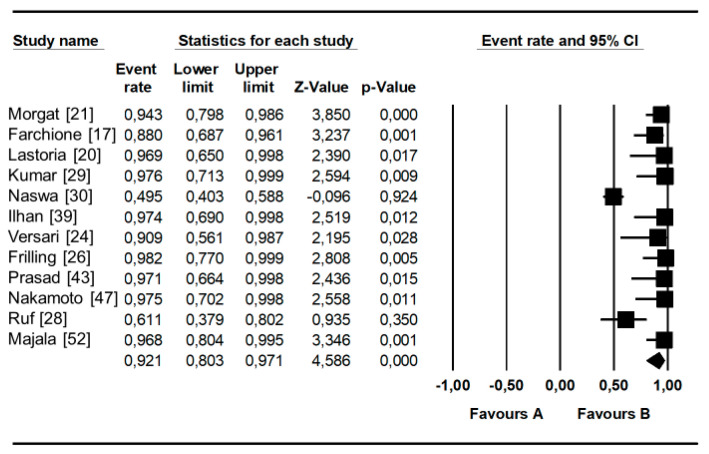
Detection rate at a per-lesion analysis. Plots of individual studies and pooled detection rate of SSR-PET/CT in patients with pNET on a per-lesion-based analysis, including 95%CI.

**Figure 4:**
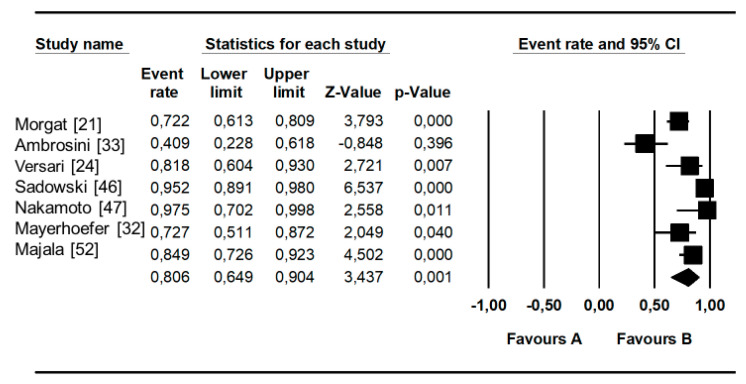
Detection rate at a per-patient analysis. Plots of individual studies and pooled detection rate of SSR-PET/CT in patients with pNET on a per-patient-based analysis, including 95%CI.

As shown in Table 3, the pooled sensitivity and specificity of SSR-PET/CT for the assessment of primary pNET were 79.6% (95%CI: 70.5–87%) and 95% (75–100%) with a heterogeneity of 59.6% and 51.5%, respectively (both *p*s = ns). Moreover, the pooled diagnostic odds ratio (DOR) value was 35.579 (95%CI: 4.673–270.90), with a heterogeneity of 21%. Pooled detection rates for the primary tumors were 81% (95%CI: 65–90%) and 92% (95%CI: 80–97%), respectively, at patient-based and lesion-based analysis (Figure 3 and Figure 4). A slight asymmetry in the forest plots was found for each analysis; therefore, publication bias may be present in the lesion-based and patient-based analyses (see also the corresponding Funnel Plots in Figure S1A,B).

## 4. Discussion

To the best of our knowledge, this is the first systematic review and meta-analysis specifically addressing the identification of the primary lesion and initial staging by using SSR-PET/CT in patients with pNETs. Several studies have used SSR-PET/CT imaging to achieve this aim, reporting variable results. However, most of these studies have limited power due to the relatively small number of enrolled patients, and the lack of specific studies precisely focused on pNETs. To derive more robust estimates on the SSR-PET/CT diagnostic accuracy in this clinical setting, we have pooled the published studies. 

Heterogeneity of selected studies may represent a limitation in a meta-analysis. Differences among patients’ selection criteria, diversity in methodological aspects, and variable study quality are the most common potential sources of bias. We detected only a moderate heterogeneity among the studies included in the pooled analysis. However, due to the relatively low number of selected studies for the quantitative analysis, subgroup analyses aiming to explain this heterogeneity was not performed. For the same reasons, subgroup analyses, including functioning or non-functioning pNETs as well as hereditary or sporadic pNETs, were not performed. 

Obtained data confirm that SSR-PET/CT represents a robust diagnostic tool in pNETs showing a high pooled true positive rate both at patient-based and lesion-based analysis, due to the low number of the observed false-negative findings. 

We observed high accuracy in the diagnosis of the primary lesion. However, when compared with similar meta-analyses conducted on mixed gastroenteropancreatic (GEP)-NET populations, a reduction in sensitivity was observed. Indeed, in the meta-analysis by Geijer and Breimer [53], which included 2105 GEP-NET patients, a pooled sensitivity of 93% (95%CI: 91–94%) was reported. Obviously, this difference may be related to the spatial resolution of PET/CT hampering the detection of smaller pancreatic lesions and the inclusion of higher histopathological grades pNETs, which might have increased the occurrence of some false-negative findings due to the lower SSR expression [8]. However, the inclusion of patients affected by insulinoma in many of analyzed studies might have also contributed. Indeed, compared to carcinoids (the commonest GEP-NET histopathological subtype), insulinomas have limited SSR expression, thus potentially reducing SSR-PET sensitivity [54]. On the other hand, pancreatic SSR expression might represent a potential source of false-positive results in the primary lesion detection by SSR-targeted molecular imaging (thus reducing specificity). Indeed, particularly the head and uncinate process, represent a site of physiological SSR overexpression [55,56,57]. Previous studies proposed a cutoff value of SUVmax for differentiating between physiologic and neoplastic pancreatic uptake [57], but there is some overlap of SUV reported in the literature for these two different conditions. This topic was assessed by three of the selected studies [31,34,37]. Overall, they showed that caution is suggested when dismissing foci of enhanced uptake seen on functional but not on anatomic imaging as false positive, especially in patients with repeat PET findings on follow-up period.

Of note, a wide heterogeneity in SUVmax values was observed between primary pancreatic lesions and distant metastases. This is coherent with the heterogeneity in SSR-2A expression between the primary site and distant metastases, since metastatic lesions, being comparatively new as compared to the primary, may be subject to less intense down-regulation of SSR [58]. This difference may theoretically impact the SSR-PET/CT diagnostic accuracy, introducing in a site-specific detection rate heterogeneity. However, contradictory findings were reported in the analyzed studies. The use of different SSR tracers, the variable sample size, the inclusion of a mixed patient population of pNET and GEP-NET, and the variability of histopathological tumor subtypes are possible reasons behind this contradictory finding. The combined use of FDG and SSR-PET/CT imaging may at least partially solve this clinical issue [59].

When compared with conventional imaging, SSR-PET/CT offered a relevant advantage in the detection rate of most metastatic sites. These additional findings have prompted therapeutic interventions in some patients, as shown by Ilhan et al. [39]. They also have a prognostic implication because unknown distant bone metastases are considered as a negative prognostic factor, possibly requiring a more aggressive treatment regime [60]. Therefore, this method is now the choice to fully stage and localize the extent of disease in patients with non-insulinoma pNETs in the preoperative setting by the current guidelines of the European Neuroendocrine Tumor Society (ENETS) [61].

Only in a few cases, the SSR-PET/CT approach was integrated with contrast-enhanced (ce) CT [32,45]. In the study by Kazmierczak et al. [45], this combination resulted in an improvement in sensitivity of 50% and an improvement in accuracy of 30% in primary tumor detection. However, Mayerhoefer et al. [32] showed that sensitivity improvement is only moderate while hardly affecting specificity, concluding that unenhanced images may be enough for routine PET/CT in NET patients. Moreover, none of these studies was conducted exclusively in pNET patients. Similarly, combining PET with MRI may take the theoretical advantage of the combination of high soft-tissue contrast for MRI with metabolic data from PET, helping to recognize small lesions. Moreover, MRI is a tool-free of ionizing radiation. However, only a few studies demonstrated a positive impact of PET/MRI in studying NETs [62,63,64,65], and evidence specifically addressing pNETs is still anecdotal [66,67]. Further studies are needed to understand better the potential role of this tool in this field.

## 5. Conclusions

SSR-PET/CT has a high detection rate and diagnostic performances for primary lesion and initial staging of pNETs. Further studies are needed to validate the integration of SSR-PET with contrast-enhanced CT or MRI in this clinical setting.

## Figures and Tables

**Table 1:** Characteristics of included studies.

Study Characteristics						Patients Characteristics					
Reference	Year	Journal	Country	Study Design	Setting	N Pancreas/N Patients	Mean Age (Range)	Grading	Type of Treatment (*n*)	Detection of Suspected pNET-Diagnosis *n*	Staging *n*
[22]	2007	J Nucl Med	Austria	prospective	diagnosis, staging, restaging	18/84	58 (28–79)	nr	6 surgery 7 surgery + chemotherapy 16 surgery + long-acting somatostatin analogs	13	36
[23]	2009	J Nucl Med	Austria	retrospective	staging and restaging	11/51	Nr (32–87)	nr	nr	0	11
[24]	2010	Clin Nucl Med	Italy	retrospective	diagnosis	11/19	56 (21–80)	9 WDET 4 WDEC	none	11	0
[25]	2010	J Nucl Med	UK	retrospective	staging, restaging	13/51	55.5	28 G1 19 G2 4 G3	9 surgery 10 Chemotherapy 27 long-acting somatostatin analogs 5 none	0	13
[26]	2010	Ann Surg	Germany	prospective	diagnosis, staging	27/52	52 (24–76)	51 G1-G2 1 G3	none	1	26
[27]	2010	EJNMMI	Germany-Italy	retrospective	diagnosis	16/59	65	16 G1 2 G2 4 G3 13 nr	none	16	0
[28]	2010	Neuro endocrinology	Germany	retrospective	diagnosis, staging, restaging	18/66	56 (29–79)	nr	33 surgery 5 surgery + chemotherapy 4 surgery + long-acting somatostatin analogs 2 long-acting somatostatin analogs 2 long-acting somatostatin analogs + chemotherapy 2 surgery + long-acting somatostatin analogs + chemotherapy 1 chemotherapy 17 none	nr	nr
[29]	2011	Eur Radiol	India	retrospective	diagnosis and staging	20/20	42.5*	nr	none	17	3
[30]	2011	AJR	India	prospective	staging, restaging	26/109	50* (21–76)	nr	60 none 30 surgery 11 surgery + long-acting somatostatin analogs 5 long-acting somatostatin analogs 2 surgery + chemotherapy or radiotherapy 1 long-acting somatostatin analogs + chemotherapy	0	26
[31]	2012	Clin Nucl Med	Israel	retrospective	diagnosis, treatment response evaluation, detection of SSTR expression	40/96	59 (16–89)	nr	Nr	0	40
[32]	2012	Eur Radiol	Austria	retrospective	diagnosis, staging, restaging	19/55	62 (37–80)	34 G1 10 G2 4 G3	nr	19	0
[33]	2012	EJNMMI	Italy	retrospective	staging, restaging	10/131	nr	nr	nr	10	0
[34]	2013	EJNMMI	Austria	retrospective	staging and restaging	22/249	59.5 (15–90)	nr	nr	nr	nr
[35]	2013	EJNMMI	Germany	retrospective	diagnosis	18/18	56 (26–80)	nr	6 surgery other nr	18	0
[10]	2013	Recent Results Cancer Res	Germany	prospective	before PRRT	9/27	62 (46–81)		7 surgery 7 surgery + chemotherapy + PRRT 5 surgery + long-acting somatostatin analogs 2 none 6 other	9	0
[36]	2014	Clin Nucl Med	Poland	retrospective	staging or restaging after surgery	56/245	56 (18–78)	G1: 103 G2: 142	35 surgery 12 PRRT 6 chemotherapy 16 long-acting somatostatin analogs	0	56
[37]	2014	Nucl Med Comm	United Kingdom	retrospective	diagnosis, restaging after surgery, treatment response evaluation, detection of SSTR expression	38/138	56 (20–84)	nr	nr	0	38
[38]	2015	Pancreas	Germany	retrospective	staging	19/19	58 (33–72)	G1: 3 G2: 15 G3: 1	Pre-operative	0	19
[19]	2015	J Am Coll Surg	USA	prospective	staging, restaging	26/26	42 (19–82)	nr	10 surgery 16 none	12	14
[39]	2015	Ann Surg Oncol	Germany	prospective	staging	18/44	56* (32–77)	nr (but all G1-G2)	Pre surgery	0	18
[40]	2015	J Nucl Med	Denmark	prospective	staging, restaging	11/59	61 (32–81)	G1: 12 G2: 40 7 nr	32 surgery ** 32 long-acting ** somatostatin analogs 27 interferon ** 19 PRRT ** 16 chemotherapy **	0	11
[41]	2015	Abdom Imaging	India	retrospective	staging, restaging	141/141	46 (6–81)	nr	nr	88	0
[42]	2015	J Nucl Med	UK	retrospective	all	142/728	54 (15–86)	260 G1 89 G2 63 G3	nr	nr	nr
[43]	2016	EJNMMI	Germany	prospective	diagnosis	20/20	45 (22–64)	nr	nr	20	0
[20]	2016	Endocrine	Italy	prospective	diagnosis	11/18	40 (16–61)	nr	nr	11	0
[17]	2016	Pancreas	Italy	retrospective	staging, treatment response evaluation	25/25	58 (27–84)	G1: 7 G2: 7 G3: 2 nr: 9	16 naive 6 SST analogs 1 PRRT1 PRRT + Chemotherapy 1 PRRT+SST analogs	0	25
[21]	2016	EJNMMI	France	prospective	diagnosis and restaging	19/19	47 (26–70)	nr	surgery	4	15
[44]	2016	J Nucl Med	USA	prospective	diagnosis, staging, treatment response	22/97 (gastro-entero-pancreatic)	54	24 G1 37 G2 6 G3 30 nr	51 long-acting somatostatin analogs other nr	0	22
[45]	2016	Eur Radiol	Germany	retrospective	diagnosis, staging	12/38	63 (34–76)	16 G1 8 G2	none	12	0
[46]	2016	JCO	USA	prospective	diagnosis	31/131	51 (19–82)	nr	none	31	0
[47]	2016	Clin Radiol	Japan	retrospective	diagnosis, staging, restaging	19/54	55 (27–81)	nr	nr	8	20
[16]	2017	J Clin Endocrinol Metab	Switzerland	retrospective	diagnosis	10/31	57.5* (21.75)	G1: 8 G2: 1	surgery	10	0
[48]	2017	Pancreas	Italy	retrospective	diagnosis	35/35	59 (41–84)	G1: 10 G2: 25	surgery	35	0
[49]	2017	EJSO	Italy	prospective	diagnosis, prognostication	124/124	55	6 G1 69 G2 5 G3	63 surgery 61 none	0	124
[50]	2018	J Formos Med Assoc	Taiwan	prospective	diagnosis	10/17	56 (24–84)	G1: 6 G2: 7 G3: 1 3 nr	None	10	0
[51]	2019	AOJNMB	Turkey	retrospective	staging metastases detection	19/38	50* (27–80)	18 G1 20 G2	nr	0	19
[18]	2019	Eur J Radiol	USA	prospective	diagnosis	36/36	46	nr	nr	36	0
[52]	2019	EJNMMI Research	Finland	prospective	diagnosis, prognostication	31/31	60 (20–83)	13 G1 8 G2 1 G3	nr	0	31

* median; ** possible combination of different therapies not explained in the paper; PRRT: peptide receptor radiation therapy; SSTR: somatostatin receptors; WDET, well-differentiated endocrine tumor; WDEC, well-differentiated endocrine cancer; PD poorly differentiated; nr: not reported.

**Table 2:** Technical features of included studies.

Reference	Device	Radiotracer (Peptide)	Activity Injected MBq Mean (Range)	Uptake Time Min Mean (Range)	PET Analysis	Semiquantitative Parameters	SUVmax Mean (Range)
[22]	PET	DOTATOC	150	20, 60, 100	visual and semiquantitative	SUVmax	nr
[23]	PET/CT	DOTATOC	150	60–90	visual	/	/
[24]	PET/CT	DOTATOC	1.5–2 MBq/Kg	60	visual	/	/
[25]	PET/CT	DOTATATE	120–200	60	visual	/	/
[26]	PET/CT	DOTATOC	120–250	60	visual and semiquantitative	SUVmax	nr
[27]	PET/CT	DOTANOC	100 (46–260)	60	visual and semiquantitative	SUVmax	18.6 (7.8–34.8)
[28]	PET & PET/CT	DOTATOC	100–120	60	visual	/	/
[29]	PET/CT	DOTATOC	132–222	30–45	visual and semiquantitative	SUVmax	12.6 (8.8–27.6) only in pancreas
[30]	PET/CT	DOTANOC	132–222	45–60	visual and semiquantitative	SUVmax	13 * (1–125)
[31]	PET/CT	DOTANOC	132 (77–196)	73 (50–120)	visual and semiquantitative	SUVmax	26 (5.5–165)
[32]	PET/CT	DOTATOC	150	90	visual	/	/
[33]	PET/CT	DOTANOC	120–185	60	visual	/	/
[34]	PET/CT	DOTATOC	119 (68–220)	87 (51–148)	visual and semiquantitative	SUVmax	34.6 only in pancreas
[35]	PET/CT	DOTATATE	200	60	visual and semiquantitative	SUVmax	36.5
[10]	PET and PET/CT	DOTATOC and DOTATATE	88 (52–111) 102 (60–123)	68 (29–162) 56 (24–161)	visual and semiquantitative	SUVmax	37.4 19.6
[36]	PET/CT	DOTATATE	156 (120–200)	60–70	visual and semiquantitative	SUVmax	24.9 only in pancreas
[37]	PET/CT	DOTATATE	117 (51–212)	24–44	visual and semiquantitative	SUVmax	32 (10–151)
[38]	PET/CT	DOTATOC or DOTANOC or DOTATATE	122 (86–149)	60	visual and semiquantitative	SUVmax; SUVmean	22.5 (5.7–100.4)
[19]	PET/CT	DOTATATE	185	60	visual and semiquantitative	SUVmax	72.8 (19,2–191)
[39]	PET/CT	DOTATATE	200	60	visual	/	/
[40]	PET/CT	DOTATOC and DOTATATE°	150 200	45 60	visual and semiquantitative	SUVmax; TBR	44.5 61.2
[41]	PET/CT	DOTANOC	132–222	45–60	visual and semiquantitative	SUVmax	14.7 (5–32.5) only in pancreas
[42]	PET/CT	DOTATATE	250	45–60	visual	/	/
[43]	PET/CT	DOTATOC	1.7 MBq/Kg	45–60	visual and semiquantitative	SUVmax	18.9 (5–65.6)
[20]	PET/CT	DOTATATE	120–220	45–60	visual and semiquantitative	SUVmax	28 (3.9–85.8) only in pancreas
[17]	PET/CT	DOTANOC	2.5 MBq/Kg	60	visual and semiquantitative	SUVmax	14.6 (4.2–82.9)
[21]	PET/CT	DOTATOC	97 (74–124)	60	visual	/	/
[44]	PET/CT	DOTATATE	196	55–93	visual	/	/
[45]	PET/CT	DOTATATE	206 (127–302)	60	visual and semiquantitative	SUVmax; tumor to spleen ratio	26.5 (5.7–77.9)
[46]	PET/CT	DOTATATE	185	60	visual and semiquantitative	SUVmax	65.4 (6.9–244)
[47]	PET/CT	DOTATOC	11–185	64 (55–75)	visual and semiquantitative	SUVmax; tumor to pancreas ratio	31.7
[16]	PET/CT	DOTATATE	185	60	visual and semiquantitative	SUVmax	nr
[48]	PET/CT	DOTATOC	1.5 MBq/Kg	60	visual and semiquantitative	SUVmax	45.6 (2–178)
[49]	PET/CT	DOTANOC	nr	nr	visual	/	/
[50]	PET/CT	DOTATOC	74–185	60	visual and semiquantitative	SUVmax; SUVmean, MTV; TLG	53.8 (23,8–96) G1 11.5 (4.1–61.8) G2–3
[51]	PET/CT	DOTATATE	2 MBq/Kg	60	visual and semiquantitative	SUVmax	18.5 Only in pancreas
[18]	PET/CT	DOTATATE	185	60	visual and semiquantitative	SUVmax	nr
[52]	PET/CT	DOTANOC	143	64	visual and semiquantitative	SUVmax	(8.7–104.7)

MTV: metabolic tumor volume; nr: not reported; TBR: tumor to background ratio; TFTV: total functional tumor volume; TLG: total lesion glycolysis; ° radio-labelled with 64Cu; * median.

**Table 3:** Results from the meta-analysis.

	Sensitivity (95%CI)			Pooled Specificity (95%CI)			LR+ (95%CI)			LR– (95%CI)			DOR (95%CI)		
	Pooled	I2 (%)	X2 (*p*)	Pooled	I2 (%)	X2 (*p*)	Pooled	I2 (%)	X2 (*p*)	Pooled	I2 (%)	X2 (*p*)	Pooled	I2 (%)	X2 (*p*)
Patients-based analysis	79.6% (71–87)	59.6%	4.95 (0.08)	95% (75–100)	51.5%	4.12 (0.13)	5.76 (1.4–24.3)	21%	2.53 (0.28)	0.201 (0.06–0.70)	64%	5.56 (0.06)	35.6 (4.67–270.9)	21%	2.52 (0.28)

LR+: positive likelihood ratio; LR–: negative likelihood ratio; DOR: diagnostic odd ratio; I2: heterogeneity.

## Supplementary Materials

The following are available online at https://www.mdpi.com/2075-4418/10/8/598/s1, Figure S1A,B Funnel Plots for publication biases at the lesion-based and patient-based analyses.
