# Supplementary figures and images for: Somatostatin Receptor PET/CT Imaging for the Detection and Staging of Pancreatic NET: A Systematic Review and Meta-Analysis
Source: Diagnostics (Basel). 2020 Aug 16;10(8):598. doi: 10.3390/diagnostics10080598 (PMC7459584; doi:10.3390/diagnostics10080598)

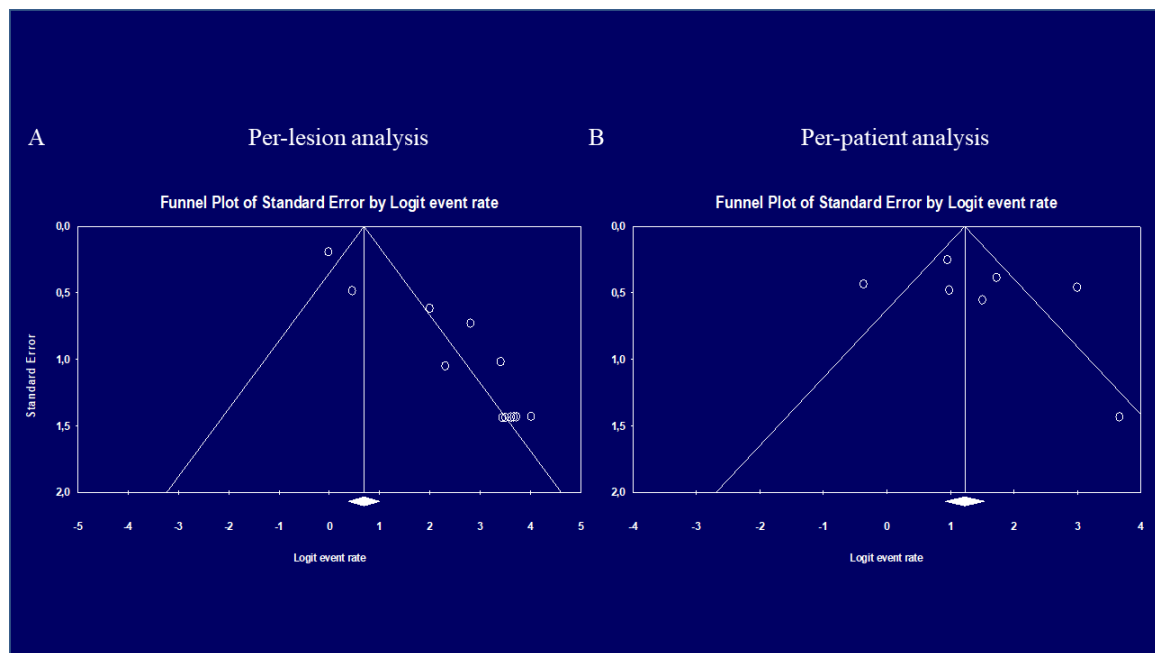

Figure S1: A-B. Funnel Plots for publication biases at the lesion-based and patient-based analyses.

Supplement: Supplementary file 1 [file diagnostics-10-00598-s001.pdf]
